# High seroprevalence of *Borrelia miyamotoi* antibodies in forestry workers and individuals suspected of human granulocytic anaplasmosis in the Netherlands

**DOI:** 10.1002/nmi2.59

**Published:** 2014-09-06

**Authors:** S Jahfari, T Herremans, A E Platonov, H Kuiper, L S Karan, O Vasilieva, M P G Koopmans, J W R Hovius, H Sprong

**Affiliations:** 1 Centre for Infectious Disease Control Netherlands, National Institute for Public Health and Environment (RIVM)P.O. Box 1, 3720 BA, Bilthoven, the Netherlands; 2 Central Research Institute of EpidemiologyMoscow, Russia; 3 FlevoziekenhuisAlmere, the Netherlands; 4 Central Research Institute of EpidemiologyMoscow, Russia; 5 Department of Internal Medicine, Section of Infectious Diseases, Section of Infectious Diseases, Academic Medical CentreAmsterdam, the Netherlands

**Keywords:** Anaplasmosis, *Borrelia miyamotoi*, *Ixodes ricinus*, relapsing fever, serology

## Abstract

Substantial exposure to *Borrelia miyamotoi* occurs through bites from *Ixodes ricinus* ticks in the Netherlands, which also transmit *Borrelia burgdorferi* sensu lato and *Anaplasma phagocytophilum*. Direct evidence for *B. miyamotoi* infection in European populations is scarce. A flu-like illness with high fever, resembling human granulocytic anaplasmosis, has been attributed to *B. miyamotoi* infections in relatively small groups. *Borrelia miyamotoi* infections associated with chronic meningoencephalitis have also been described in case reports. Assuming that an IgG antibody response against *B. miyamotoi* antigens reflects (endured) infection, the seroprevalence in different risk groups was examined. Sera from nine out of ten confirmed *B. miyamotoi* infections from Russia were found to be positive with the recombinant antigen used, and no significant cross-reactivity was observed in secondary syphilis patients. The seroprevalence in blood donors was set at 2.0% (95% CI 0.4–5.7%). Elevated seroprevalences in individuals with serologically confirmed, 7.4% (2.0–17.9%), or unconfirmed, 8.6% (1.8–23%), Lyme neuroborreliosis were not significantly different from those in blood donors. The prevalence of anti-*B. miyamotoi* antibodies among forestry workers was 10% (5.3–16.8%) and in patients with serologically unconfirmed but suspected human granulocytic anaplasmosis was 14.6% (9.0–21.8%); these were significantly higher compared with the seroprevalence in blood donors. Our findings indicate that infections with *B. miyamotoi* occur in tick-exposed individuals in the Netherlands. In addition, *B. miyamotoi* infections should be considered in patients reporting tick bites and febrile illness with unresolved aetiology in the Netherlands, and other countries where *I*. *ricinus* ticks are endemic.

## Introduction

*Borrelia miyamotoi* belongs to the relapsing fever group of the *Borrelia* genus 1. Agents of relapsing fever spirochaetes are transmitted between vertebrates by different vectors; for example, *Borrelia duttonii* and *Borrelia hermsii* are transmitted by soft ticks and *Borrelia recurrentis* is transmitted by the human body louse 2–4. Interestingly, *B. miyamotoi* is transmitted by the same vectors as *Borrelia burgdorferi* senso lato and *Anaplasma phagocytophilum*, the causative agents of Lyme borreliosis (LB) and human granulocytic anaplasmosis (HGA), respectively 4,5. In Europe, Asia and North America, *B. miyamotoi* infection rates in *Ixodes persulcatus*, *Ixodes scapularis*, *Ixodes pacificus* and *Ixodes ricinus* range between 0.5% and 5% 1,5–10. In the Netherlands, the infection rate of *I. ricinus* is 2.4–4.7% in all three life stages 11. The presence of *B. miyamotoi* in wild rodents indicates enzootic circulation in the Netherlands (S. Jahfari, unpublished observations). Furthermore, a recent study estimated that in the Netherlands annually, approximately 200 000 people are bitten by ticks infected with *B. burgdorferi* s.l. and 36 000 by *B. miyamotoi*-infected ticks 11. Additionally, exposure to both Lyme and relapsing fever *Borrelia* by co-infected ticks occurs in at least 9000 people annually. This substantial exposure raises the question to what extent *B. miyamotoi* leads to human disease in the general population.

Currently, the clinical symptoms of *B. miyamotoi* infections are undefined, and validated supportive laboratory diagnostic tests are lacking. Infections with other members of the relapsing fever *Borreliae* are characterized by flu-like illness and one or more relapse episode(s) of bacteraemia and fever. *Borrelia miyamotoi* infections in humans were first reported in Russian patients suspected of LB (Table1). Fifty-one patients with suspected LB had amplifiable *B. miyamotoi* DNA in venous blood samples, and most tested positive by commercial IgM and IgG serology assays used for LB diagnosis. This test consisted of a mixture of whole cell antigens from *Borrelia afzelii*, *B. burgdorferi* and *Borrelia garinii* 12. A potentially severe complication of *B. miyamotoi* infection is meningoencephalitis. The first American and European meningoencephalitis case reports for well-documented *B. miyamotoi* infection were described in severely immunocompromised patients 13,14 and *B. miyamotoi* was detected in cerebrospinal fluid with the use of microscopy and PCR assays. In another study, two cases of *B. miyamotoi* infection were initially mistaken for HGA 15 on the basis of their clinical manifestations after a tick bite (Table1). Disease caused by an infection with *B. miyamotoi* may be confused with other tick-borne pathogens, either because of its comparable symptoms or because of misinterpretation of a serological reaction against an (endured) co-infection.

**Table 1:** Case reports of *Borrelia miyamotoi* infections associated with disease

Reference	Methods	Patient description	Erythema migrans	Clinical manifestations
Russia 12	PCR, IgM-positive	Individuals admitted to hospital, suspected for tick-borne infection (*n* = 51)	4/51	Fever, headache, chills, fatigue, vomiting and myalgia
USA 21	Seroconversion	Previously healthy individuals (*n* = 3)	2/3	Fever, chills, sweats, headache, neck stiffness, fatigue, myalgias, arthralgias, abdominal pain, a cough, a sore throat and right inguinal lymphadenopathy
USA 13	PCR, microscopy	80-year women, (treated) non-Hodgkin's lymphoma	0/1	Meningoencephalitis, progressive decline in mental status, wobbling gait and difficulty hearing, weight loss
USA 15	PCR	61-year-old male, anorexia	Not mentioned	Severe frontal headaches, photophobia, myalgia and arthralgia, pain across the chest, muscles were tightening, sweats and episodes of fever with shaking chills
USA 15	PCR	87-year-old male, previously healthy	0/1	Severe fatigue, malaise, short of breath with activities, chills, fever and loss of appetite
the Netherlands 14	PCR, microscopy	70-year-old male, (treated) B-cell non-Hodgkin's lymphoma stage IV	Not mentioned	Meningoencephalitis, complaints of confusion, altered personality and a disturbed gait, unexplained chronic diarrhoea and bradyphrenic

Patient categories that have an (endured) infection with this relapsing-fever spirochaete are expected to have a higher seroprevalence than the general population. For other relapsing fever *Borreliae* the majority of antibodies are directed towards the variable major proteins (VMP) 16. However, antibodies to VMP have been shown to be cross-reactive to *B. burgdorferi* s.l. antigens. Furthermore, VMP is a highly variable protein that could give false-negative results in serological tests 16. On the other hand, glycerophosphodiester phosphodiesterase (GlpQ) appears to be highly conserved among all members of the relapsing fever *Borreliae*, including *B. miyamotoi*, but distinct for the spirochaetes causing LB and their near relatives. In addition, GlpQ is immunogenic in humans and shows negative results when testing sera from LB and syphilis patients 16–20. More recently, an American study showed that serum samples from 1% to 3% of residents of New England were reactive in an experimental serological assay targeting the *B. miyamotoi* GlpQ antigen 21.

The long-term objective of our studies is to gain more insight into the public health risk of *B. miyamotoi*. As a first attempt to describe the exposure of *B. miyamotoi* in the Netherlands, using a newly developed serological assay based on the GlpQ antigen, we determined here the seroprevalence of anti-*B. miyamotoi* antibodies in different risk groups within the general population. Apart from important epidemiological insights, our findings will facilitate the future identification of the clinical symptoms of *B. miyamotoi* infections and might serve as a starting point for further development of serological assays.

## Materials and Methods

### Antigen preparations and biochemicals

A DNA sequence encoding for the *B. miyamotoi* GlpQ protein was amplified from an *I. ricinus* lysate, cloned, sequence-verified, expressed and purified from an *Escherichia coli* construct (Scottish Biomedical, Glasgow, UK). Purified GlpQ was coupled to activated carboxylated microspheres by using a two-step carbodiimid reaction with an antigen to bead ratio of 50 μg/6.25 × 10^6^ 22. The beads were incubated in the dark under constant rotation at 25 rpm for 2 h at room temperature. The beads were washed three times with phosphate-buffered saline (PBS) and stored in 500 *μ*L PBS containing 0.05% (weight/volume (w/v)) sodium azide and 1% (w/v) bovine serum albumin at 4°C in the dark until used 23–25. Biochemicals and reagents used were from Sigma (St Louis, MO, USA), Pierce (Rockford, IL, USA), Bio-Rad Laboratories (Hercules, CA, USA) and Merck (Darmstadt, Germany) and were used in the highest purity available.

### Serum samples

Sera from 150 blood donors were used as negative controls to determine the background in the general Dutch population. Ten sera of PCR-confirmed *B. miyamotoi* patients were used as positive controls and were described previously 12. These sera were also positive in a anti-GlpQ serological assay developed and performed in Russia (not shown). Sera from patients infected with *Treponema pallidum* were included to test for possible cross-reactivity 26. In total, 120 serum samples from forestry workers were used as a group with high exposure to tick bites 27,28. Serum specimens from Lyme neuroborreliosis (LNB) and HGA suspected patients were obtained from the residuals of sera submitted to the National Institute for Public Health and Environment (RIVM) for routine microbiological investigations, provided the patients did not object to this use by indicating this on the diagnostic request form. Sera with no or doubtful epidemiological data and repetitive sera were excluded. Selected sera were divided in different patient groups: 54 individuals with serologically confirmed LNB based on the detection of local antibody production in the paired liquor sample and 35 individuals with serologically unconfirmed LNB. In addition, 130 samples from patients sent in to our laboratory for HGA serology, but that tested negative for IgG-specific and IgM-specific HGA, were also examined. Serological confirmation for LB was based on positive test results of *B*. *burgdorferi* s.l. Immunoblot (in house), and C6-ELISA (Immunetics, Boston, MA, USA). Immunofluorescence assays against *Anaplasma phagocytophylum* and *Ehrlichia chaffeensis* (Focus Technologies, Cypress, CA, USA) were used. ‘Serological unconfirmed’ was defined as negative test results in these serological assays.

### Serological analysis

Antibodies to the GlpQ protein of *B. miyamotoi* were determined by an in-house Luminex assay. Serum dilutions and conjugate concentrations in the Luminex assay were optimized using checkerboard titrations. Sera and positive and negative control samples were tested in duplicate or triplicate and were diluted 1 : 200 in 25 μL PBS containing 0.1% (v/v) Tween-20 and 3% (w/v) bovine serum albumin. Serum samples were mixed with an equal volume of GlpQ-conjugated microspheres (4000 beads/region/well) in a 96-well Multiscreen HTS filter plate (Millipore Corporation, Billerica, MA, USA) and incubated on a plate shaker at 600 rpm in the dark for 45 min at room temperature. Blank and control sera were included on every plate. The beads were collected by filtration using a vacuum manifold and washed three times with 100 μL PBS. To each well, 50 μL of a 1 : 200 dilution of R-phycoerthyryn-conjugated goat anti-human IgG (Jackson ImmunoResearch Laboratories, Westgrove, PA, USA) in PBS was added and the plate was incubated for 30 min with continued shaking. After a second wash, the beads were resuspended in 100 μL PBS and shaken before analysis with a Bio-Plex 200 in combination with Bio-Plex Manager software version 4.1.1 (Bio-Rad Laboratories).

### Statistical analysis

The Kolmogorov–Smirnov test was used to test null hypotheses that the LogOD values of different groups were normally distributed. Fisher's exact test was used to calculate the 95% CI. A one-tailed Fisher's exact test was used to compare pairwise the frequency of *B. miyamotoi* seropositive and seronegative subjects in different groups. The level of significance was set at p <0.05. Moreover, Taylor series was used to calculate OR with a 95% CI testing.

## Results

All serum samples (*n* = 513) were tested for their reactivity against the GlpQ protein of the European *B. miyamotoi*. The LogOD values of the samples from 150 blood donors were normally distributed (p 0.56), whereas the LogOD values of all samples from the risk groups were not (p <0.004), indicating that the latter distribution is the mixture of two distributions (not shown). To strive for a relatively high specificity, a cut-off value of two standard deviations above the average of the blood donor group (*n* = 150) was chosen. This is in line with recommendations by the CDC to confirm the diagnosis of tick borne relapsing fever in the USA. With this chosen cut-off (LogOD 3.50), the seroprevalence of *B. miyamotoi* IgG antibodies in blood donors was 2.0% (Table2) and nine out of the ten sera from confirmed *B. miyamotoi*-infected patients from Russia were also serologically positive. Only one out of 24 serum samples from patients infected with an unrelated spirochaete, *Treponema pallidum*, showed reactivity against the GlpQ antigen, and the seroprevalence in this category was comparable to that of the blood donors. Although an increase in seroprevalence was observed comparing blood donors to confirmed and unconfirmed LNB patients, these seroprevalences did not differ significantly from that of the blood donors (Table2). In the serologically unconfirmed but suspected HGA group a significantly higher seroprevalence was observed compared with that in the blood donor panel. In addition, the seroprevalence in forestry workers was significantly higher than the seroprevalence in blood donors (Table2).

**Table 2:** Seroprevalence using *Borrelia miyamotoi* GlpQ

Panels of serum samples, logOD = 3.50	Tested (*n*)	Positive (*n*)	Positive (%)	Fisher exact 95% confidence	Difference from blood donors, p-value (one-tailed), Fisher exact test	OR	95% CI
Blood donors (background)	150	3	2.0%	0.4–5.7%	NA	NA	
Forestry workers (high risk)	120	12	10%	5.3–16.8%	**0.005**	**5.44**	**1.5–19.76**
LNB; serologically confirmed	54	4	7.4%	2.0–17.9%	0.08	3.92	0.85–18.12
LNB; serologically unconfirmed	35	3	8.6%	1.8–23.0%	0.08	4.60	0.89–23.81
HGA; serologically unconfirmed	130	19	14.6%	9.0–21.8%	**<0.001**	**8.40**	**2.42–29.05**
*Treponema pallidum* infection, confirmed	24	1	4.2%	0.2–18.9%	0.45		

Significant p-values (Fisher's exact test, level of significance was set at p <0.05) and OR (Taylor series, with 95% CI) in bold.

Abbreviations: GlpQ, Glycerophosphodiester phosphodiesterase; NA, not applicable; LNB, Lyme neuroborreliosis; HGA, human granulocytic anaplasmosis.

## Discussion

This study provides serological evidence of *B. miyamotoi* exposure in humans in the Netherlands using the GlpQ antigen of *B. miyamotoi* in a newly developed assay. Based on the significantly higher anti-*B. miyamotoi* antibodies in forestry workers and the serologically unconfirmed but suspected HGA group, *B. miyamotoi* infection appears not only to occur in immunocompromised patients, as has recently been described in the Netherlands 14, but also seems to affect populations without underlying or chronic disease, which is in line with recent studies 12,15,21.

The seroepidemiology of the prevalence of IgG antibodies against *B. miyamotoi* as evidence for (endured) infection was examined in this study. Therefore, the study was set up to determine the seroprevalence in the different populations at a given moment, a cross-sectional design, determining *B. miyamotoi* exposure rather than infection. No discrimination between endured or active infection was made, we did not determine a rise, decline or persistence of specific antibody production in these individuals nor did we determine the presence of anti-*B. miyamotoi* IgM antibodies. According to the CDC, to confirm the clinical diagnosis of other tick-borne relapsing fevers caused by *B. hermsii*, *Borrelia parkerii* or *Borrelia turicatae*, specific antibody titres should increase fourfold between acute and convalescent serum samples. Because of the cross-sectional design of this study, no convalescent serum samples were tested and therefore we were not able to assess the course of the serological response over time. Patients with other tick-borne relapsing fevers may have false-positive tests in indirect immunofluorescence assay or whole lysate ELISA for LB because of the similarity of proteins between the two organisms. Because of the inherent disadvantages of cultivation and PCR-based methods as diagnostic tools, a serological test such as the one we describe here is an important step towards a new diagnostic tool.

Our aim was to identify risk groups with significantly more exposure than the general population. As recommended by the CDC we used a cut-off two standard deviations above the average of the blood donor group in order to strive for a relatively high specificity. This is higher than the mathematical optimal cut-off value (LogOD 3.45), which gives the best discrimination between positive and negative serum samples, yet without taking false-positivity into account (data not shown). Nonetheless, assays for exposure to *B. miyamotoi*, such as described here and for other recombinant GlpQ antigen ELISA and immunoblots described before 21, need to be more extensively validated and tested to determine the specificity and sensitivity. In the Netherlands, we have not identified another relapsing fever spirochaete other than *B. miyamotoi* in ticks obtained from the environment, so far. Therefore, we assume that cross-reactivity between different relapsing fever GlpQ 16 has no, or an insignificant, influence in the Dutch setting. In addition, no significant cross-reactivity was observed with *T. pallidum*-positive patients.

An increased seroprevalence was observed in unconfirmed and confirmed LNB patients, which might be attributed to exposure to ticks infected with both *B. burgdorferi* s.l. and *B. miyamotoi* 11,14. Whether co-infection with *B. miyamotoi* exists and may alter the clinical manifestations of LB or LNB remains to be answered, our serological evidence suggest that it can occur in the same patients, assuming that patients were diagnosed correctly. From our data we cannot conclude whether these were concomitant or serial infections. It should be mentioned that, in analogy to *B. burgdorferi* s.l. infection in Europe, it might also be that anti-*B. miyamotoi* antibodies are a result of a previous asymptomatic infection. To answer these questions a longitudinal approach would be required.

Previous studies conducted in the Netherlands showed that forestry workers have an increased risk of acquiring infections transmitted by ticks 27–30. In this study, 11.6% (6.5–18.8%) of the forestry workers tested positive for anti-*B. miyamotoi* IgG antibodies. These findings indicate that in addition to LB and other tick-borne pathogens, forestry workers also run an occupational risk of acquiring infection with *B. miyamotoi* or at least being exposed to this tick-borne pathogen. Furthermore, based on the findings presented here, demonstrating that the group of patients with suspected HGA have a significantly higher seropositivity compared with blood donors, it is suggested that *B. miyamotoi* might also cause a febrile illness comparable to HGA. This is in line with previous case reports 15. Indeed, the clinical manifestations of HGA might be similar to those caused by *B. miyamotoi* infection, including (high-grade) fever, chills, myalgia, nausea and headache a few weeks after a tick bite. Therefore, clinicians should include *B. miyamotoi* in the differential diagnosis of patients with a febrile illness caused by an unknown aetiological agent when there is evidence of tick exposure.

In conclusion, a Luminex-assay was developed for seroepidemiological screening of IgG antibodies against the GlpQ protein of *B. miyamotoi*. We observed an increase in seroprevalence from blood donors compared with unconfirmed and confirmed LNB patients. However, only the forestry workers and the serologically unconfirmed but suspected HGA group consistently showed significantly higher seroprevalence compared with the blood donor panel. It could be that some patients identified as having LB and definitely some that were suspected of having HGA, substantiated in part by non-specific serological tests such as a whole-cell ELISA or not substantiated at all, may actually have been infected with these relapsing fever spirochaetes. Furthermore, our study suggests that individuals that are (occupationally) exposed to ticks in the Netherlands, such as forestry workers, are potentially at risk for *B. miyamotoi* infection. Interestingly, in light of the popularity of outdoor recreational activities among Dutch people, it is to be expected that this will certainly predispose a large number of people to the risk of infection with *B. miyamotoi*, among other tick-borne pathogens. Therefore, *B. miyamotoi* infection should be included in the differential diagnosis when forestry workers or patients who engage in outdoor recreational activity present with fever after a tick bite. In addition, in patients with unexplained fever or HGA-like symptoms after exposure to ticks, physicians should consider *B. miyamotoi* infection. Until better validated diagnostic tests become available it should be recommended to consult academic referral centres when there is suspicion of *B. miyamotoi* infection.
